# National Board of Health vs. Louisiana State Board of Health

**Published:** 1880-11-20

**Authors:** 


### *NATIONAL BOARD OF HEALTH VS. LOUISIANA STATE BOARD OF HEALTH.*

We notice in the New Orleans *Picayune* that at a recent meeting of the Louisiana State Board of Health quite a heated discussion was held touching a communication, published in one of the city papers, by Dr. Bemiss, a resident physician of New Orleans, and member of the National Board of Health, in which he reflected severely upon the State Board.

The communication was denounced as false in many of its representations, and the following resolution introduced:

WHEREAS, The representative of the National Board of Health, Dr. S. M. Bemiss, has thought proper, (through the columns of the New Orleans Medical Journal, of which he is an editor), to unjustly assail and grossly misrepresent the State Board of Health of Louisiana and its honored President: be it therefore

*Resolved*, That the President of this Board be and he is hereby respectfully requested to officially reply to the aforesaid article, and to present to the people of this State, through the columns of our newspapers, a clear and comprehensive statement of all the facts in controversy.

President Joseph Jones stated that he was at a loss to know how to regard the communication of the member of the National Board of Health, published in the Democrat of yesterday morning, and said to be an editorial printed in the New Orleans Medical and Surgical Journal, edited by S. M. Bemiss, M.D., W. H. Watkins, M.D., and S. S. Herrick, M.D.—whether as a farcical travesty upon the National Board of Health, or a malignant effort to injure the State Board of Health, and to excite the prejudices and animosities of the States lying above and around Louisiana.

We have not space to add the other remarks of President Jones on the subject, and have not before us the other side of the question. The outside profession not being familiar with all the facts and surroundings, cannot easily decide upon the merits of the controversy, and will be likely to view with regret this want of harmony between two high professional bodies, while the public, we fear, will be inclined to regard such discussion as not very well comporting with the dignity of the profession, and the high character of the parties to the controversy. W.

